# Procalcitonin and Interleukin-10 May Assist in Early Prediction of Bacteraemia in Children With Cancer and Febrile Neutropenia

**DOI:** 10.3389/fimmu.2021.641879

**Published:** 2021-05-20

**Authors:** Marcel Doerflinger, Gabrielle M. Haeusler, Connie S. N. Li-Wai-Suen, Julia E. Clark, Monica Slavin, Franz E. Babl, Zoe Allaway, Francoise Mechinaud, Gordon K. Smyth, Richard De Abreu Lourenco, Bob Phillips, Marc Pellegrini, Karin A. Thursky

**Affiliations:** ^1^ Infectious Diseases and Immune Defence Division, Walter and Eliza Hall Institute for Medical Research, Parkville, VIC, Australia; ^2^ Department of Medical Biology, The University of Melbourne, Melbourne, VIC, Australia; ^3^ Department of Infectious Diseases, Peter MacCallum Cancer Centre, Melbourne, VIC, Australia; ^4^ NHMRC National Centre for Infections in Cancer, University of Melbourne, Melbourne, VIC, Australia; ^5^ Sir Peter MacCallum Department of Oncology, University of Melbourne, Melbourne, VIC, Australia; ^6^ Infection and Immunity Theme, Murdoch Children’s Research Institute, Parkville, VIC, Australia; ^7^ Infection Diseases Unit, Department of General Medicine, Royal Children’s Hospital, Parkville, VIC, Australia; ^8^ Queensland Children’s Hospital, Child Health Research Centre, University of Queensland, Brisbane, QLD, Australia; ^9^ Department of Medicine, University of Melbourne, Melbourne, VIC, Australia; ^10^ Victorian Infectious Diseases Service, The Peter Doherty Institute for Infection and Immunity, Melbourne, VIC, Australia; ^11^ Emergency Department, Royal Children’s Hospital, Parkville, VIC, Australia; ^12^ Paediatric Emergency Medicine Centre of Research Excellence, ED Research Group, Murdoch Children’s Research Institute, Parkville, VIC, Australia; ^13^ Department of Paediatrics, Faculty of Medicine, Dentistry and Health Sciences, University of Melbourne, Melbourne, VIC, Australia; ^14^ Unité D’hématologie Immunologie Pédiatrique, Hopital Robert Debré, APHP Nord Université de Paris, Paris, France; ^15^ School of Mathematics and Statistics, The University of Melbourne, Melbourne, VIC, Australia; ^16^ Centre for Health Economics Research and Evaluation, University of Technology Sydney, Sydney, NSW, Australia; ^17^ Leeds Children’s Hospital, Leeds General Infirmary, Leeds, United Kingdom

**Keywords:** cancer, children, febrile neutropenia, biomarkers, risk stratification

## Abstract

**Objectives:**

Febrile neutropenia (FN) causes treatment disruption and unplanned hospitalization in children with cancer. Serum biomarkers are infrequently used to stratify these patients into high or low risk for serious infection. This study investigated plasma abundance of cytokines in children with FN and their ability to predict bacteraemia.

**Methods:**

Thirty-three plasma cytokines, C-reactive protein (CRP) and procalcitonin (PCT) were measured using ELISA assays in samples taken at FN presentation (n = 79) and within 8–24 h (Day 2; n = 31). Optimal thresholds for prediction of bacteraemia were identified and the predictive ability of biomarkers in addition to routinely available clinical variables was assessed.

**Results:**

The median age of included FN episodes was 6.0 years and eight (10%) had a bacteraemia. On presentation, elevated PCT, IL-10 and Mip1-beta were significantly associated with bacteraemia, while CRP, IL-6 and IL-8 were not. The combination of PCT (≥0.425 ng/ml) and IL-10 (≥4.37 pg/ml) had a sensitivity of 100% (95% CI 68.8–100%) and specificity of 89% (95% CI 80.0–95.0%) for prediction of bacteraemia, correctly identifying all eight bacteraemia episodes and classifying 16 FN episodes as high-risk. There was limited additive benefit of incorporating clinical variables to this model. On Day 2, there was an 11-fold increase in PCT in episodes with a bacteraemia which was significantly higher than that observed in the non-bacteraemia episodes.

**Conclusion:**

Elevated PCT and IL-10 accurately identified all bacteraemia episodes in our FN cohort and may enhance the early risk stratification process in this population. Prospective validation and implementation is required to determine the impact on health service utilisation.

## Introduction

Infection, frequently presenting as fever and neutropenia (FN), is the leading cause of unplanned hospital admissions for administration of broad spectrum antibiotics in children with cancer (1). There is a growing international effort to optimise the management of children with cancer and FN through the use of risk stratification strategies (2). This is in recognition of the heterogenous nature of FN and the data to support that a large proportion of children with FN are low-risk of severe infection or adverse outcome (2, 3).

Early de-escalation of standard inpatient care and cessation of intravenous antibiotics in patients identified as low-risk has been shown to reduce antibiotic exposure and hospital length of stay and improvement in quality of life (4–6). Ensuring these management pathways have the greatest impact without compromising patient safety, relies on the early (<24 h) and accurate identification of children with low-risk FN (7). To date, much of the attention has focused on the derivation and validation of decision rules incorporating readily available laboratory and clinical parameters to predict infection or adverse outcome and the role of novel biomarkers in augmenting these rules, remains unknown (8).

Studies in children with cancer and FN have explored the value of a range of inflammatory cytokines in isolation in predicting infection and complications (9, 10). An updated systematic review found that interleukin (IL)-6, IL-8, C-reactive protein (CRP) and procalcitonin (PCT) were predictive of bacteraemia and severe sepsis as compared to a range of other biomarkers however the optimal thresholds in FN remain unknown (11). While the role of serial biomarkers has also been explored, the few studies that have investigated the diagnostic performance of cytokines at presentation and Day 2 have mixed results (11).

The objectives of this study were to compare and contrast the ability of an extended range of 34 serum cytokines, in addition to more commonly available PCT and CRP, to predict bacteraemia in children with cancer and FN. We also explored the trend of these biomarkers on Day 2 as well as the additional predictive value these biomarkers have over or in combination with routinely available clinical data.

## Materials and Methods

### Patient Recruitment and Blood Sample Collection

This prospective biomarker study was embedded into the Australian Predicting Infectious ComplicatioNs In Children with Cancer (PICNICC) study (Australian New Zealand Clinical Trials Registry 12616001440415) (12). The Australian PICNICC study is a large, multisite, prospective study designed to validate existing paediatric FN clinical decision rules (CDRs) and identify novel biomarkers that predict severe infection (12, 13). Patients were enrolled at two of the eight study sites: Royal Children’s Hospital (RCH), Melbourne and Queensland Children’s Hospital (QCH), Brisbane.

Children with solid-organ cancer or leukaemia on active treatment and who presented to the emergency department with FN were included. Multiple, discrete FN episodes per patient were allowed. Fever was defined as a single tympanic temperature ≥38 °C and neutropenia was defined as an absolute neutrophil count (ANC) <1,000/mm^3^. Children with hematopoietic stem cell transplant (HSCT) within three months and those receiving treatment antibiotics were excluded. Demographic, FN episode and outcome data were prospectively collected by the site research assistant (RA) from electronic and paper-based records and entered into research electronic data capture (REDCap).

To optimise patient recruitment, the electronic medical record (Epic) was utilised at one site (RCH). A Best Practice Alert (BPA) was developed that identified cancer patients on active treatment and who presented to the emergency department with fever and who had a valid RCH Children’s Cancer Centre (CCC) tissue bank patient consent. The BPA fired when the treating clinician ordered blood samples and included the following statement: “This patient has consented to provide samples to the CCC Tissue Bank and presents with possible febrile neutropenia—please order the required CCC Tissue Bank sample.” An option for not-eligible or declined sample was included on the alert.

Blood from eligible patients was collected at two time points: FN onset (within 0–4 h of ED presentation and prior to the first dose antibiotic) and Day 2 (within 8–24 h of ED presentation). Blood was collected in EDTA tubes and processed by the cancer centre’s tissue banks at RCH and QCH according to a standardised protocol. Within two hours of sample collection, plasma was separated from erythrocytes and peripheral blood mononuclear cells (PBMCs) using Ficoll density gradient centrifugation and stored at −80°C until thawed for biomarker analysis.

The primary outcome was bacteraemia and defined according to paediatric FN research consensus definitions (see **Table 1**) (16). All infection diagnoses were made from clinical symptoms or microbiological samples taken within 48 h of FN onset. Microbiological investigations were performed according to site FN guidelines. Across both sites this included: at least one blood culture set (for all patients, and before antibiotic treatment commenced) and urine for culture; nasal swab for respiratory virus PCR; chest X-ray; stool for culture, *Clostridioides difficile* toxin assay and viral PCR; and skin or wound swab for culture and viral PCR (as indicated) (**Table 1**).

**Table 1:** Definitions of outcomes included in analysis.

Outcome	Definition
**Bacteraemia** (14)	A recognised bacterial pathogen (including organisms associated with mucosal barrier injury in the setting of mucositis or neutropenia) from ≥1 blood culture set or common commensals from ≥2 blood culture sets drawn on separate occasions.
**Microbiologically documented infection (MDI)** (14)	An infection that was clinically detectable and microbiologically proven.
**Clinically documented infection (CDI)** (14)	A site of infection that is diagnosed but its microbiological pathogenesis either cannot be proven or is inaccessible to examination.
**Fever of unknown origin (FUO)** (14)	Any febrile episode without a clinically detectable and microbiologically proven infection.
**AUS-rule** (15)	A paediatric FN clinical decision rule that incorporates three clinical variables (platelets <50 g/L, total white cell count <300 cells/m^3^ and chemotherapy more intensive than acute lymphoblastic leukaemia-maintenance phase) each with a weighted value of ‘1’ to predict bacterial infection. AUS-rule scores range from 0 to a maximum of 3.
**End of FN episode**	Afebrile for more than 48h, recovery of ANC beyond nadir and antibiotic cessation.

The additive predictive ability of biomarkers to routinely available clinical variables was assessed. Clinical variables previously identified to be predictive of bacterial infection in the Australian PICNICC dataset were used and included: height of temperature, platelet count and clinically unwell (13). Clinically unwell was defined as severe sepsis or septic shock (as per Goldstein et al.) (15), altered conscious state (Glasgow Coma Score <15 or only responsive to voice or pain), documented as ‘severely unwell’ or equivalent in patient record or either blood pressure or respiratory rate within the Victorian age-based mandatory emergency call range (12).

The additive predictive ability of the commercially available PCT to the recalibrated CDR that showed the best performance in the Australian PICNICC study dataset (AUS-rule) was also determined (13). The AUS-rule uses three clinical variables (platelets <50 g/L, total white cell count <300 cells/m^3^ and chemotherapy more intensive than acute lymphoblastic leukaemia-maintenance phase) each with a weighted value of ‘1’ to predict bacterial infection in children with cancer and FN. The rule has been validated internationally (14) and is being implemented across Australia (ACTRN12616001440415).

### Blood Plasma Biomarker Analysis

#### Procalcitonin (PCT) and C-Reactive Protein (CRP) Analysis

PCT in patient plasma was quantified using the COBAS Elecsys BRAHMS electrochemiluminescence immunoassay “ECLIA” on the COBAS e 801 immunoassay analyzer. CRP was quantified using VITROS Chemistry Products CRP Slides and the VIRTOS 250/350 system according to the manufacturers instruction.

#### Multiplex Plasma Cytokine ELISA Analysis

The Human Cytokine & Chemokine 34-plex ProcartaPlex 1A multiplex immunoassay kit (Invitrogen/Thermo #EPX340-12167-901) was used to assay the abundance of the following plasma cytokines: Eotaxin/CCL11; GM-CSF; GRO alpha/CXCL1; IFN alpha; IFN gamma; IL-1 beta; IL-1 alpha; IL-1RA; IL-2; IL-4; IL-5; IL-6; IL-7; IL-8/CXCL8; IL-9; IL-10; IL-12 p70; IL-13; IL-15; IL-17A; IL-18; IL-21; IL-22; IL-23; IL-27; IL-31; IP-10/CXCL10; MCP-1/CCL2; MIP-1 alpha/CCL3; MIP-1 beta/CCL4; RANTES/CCL5; SDF1 alpha/CXCL12; TNF alpha; TNF beta. As per manufacturers instruction, 25 µl of each sample were thawed and stained with the respective cytokine bead mix and samples run on the Bio-plex 200 system (Bio-Rad Laboratories, USA). Data for a total of 33 cytokines was obtained, however, TNF beta ELISA measurements could not be obtained. Cytokine concentrations were calculated using Bio-Plex Manager 5.0 software (Bio-Rad Laboratories, USA) with a five-parameter curve-fitting algorithm applied for standard curve calculations.

### Statistical Analysis

To determine if the subset of patients with a blood sample taken is representative of the entire RCH and QCH cohort the following characteristics were compared: median age, sex, diagnosis, infective diagnosis, severe sepsis, intensive care unit (ICU) admission, median length of stay (LOS), death. The Youden and Liu method for cut-point analysis of receiver operator curves (ROC) was used to define the value that best predicts bacteraemia for each plasma biomarker molecule by optimally balancing sensitivity and specificity. Predictive cut off values as well as positive and negative predictive values were calculated based on this Youden ROC analysis. Analyses were performed using Graphpad prism v8.

A classification tree was constructed to identify patients with bacteraemia from the serum cytokine measurements and clinical data (17). To increase sensitivity and to balance the influence of bacteraemia vs non-bacteraemia episodes, a loss matrix was incorporated to weight the cost of misclassification for false negatives as 10 times more than that for false positives. To avoid very small partitions, the minimum number of observations in a node was set to 10. The estimated decision tree was pruned based on a complexity parameter of 0.01. In order to estimate the predictive misclassification rate, the final model was validated using cross-validation whereby each patient was excluded from the data in turn to train the model on the remaining patients and predictions made for the observations from that patient. The optimal recursive partitioning of the predictor variables was estimated using the rpart package (18) in R 4.0.0 (19).

## Results

### Characteristics of FN Episodes

Blood samples for cytokine analysis were taken at presentation (between 0 and 4 h) in 80 out of 553 FN episodes, enrolled at RCH and QCH as part of the Australian PICNICC study (RCH n = 68; QCH n = 12). Sufficient serum for analysis was unavailable for one patient leaving 79 samples occurring in 64 patients (12 patients had two FN episodes, two patients had three FN episodes). In 31 episodes, a second sample was taken at Day 2 (between 8 and 24 h from presentation). The median time between Presentation and Day 2 sample was 21.5 h (IQR: 18.25–23.3 h, range 6.83–30.25). All FN episodes had blood cultures collected prior to the first dose antibiotic.

The median age of patients with a blood sample taken was 6.0 years, slightly higher than those without a sample taken. Otherwise there was no significant difference in baseline demographics, causes of infection and clinical complications between the FN episodes with and without a blood sample taken (**Table 2**), as well as between episodes with blood sample taken at Days 1 and 2 (data not shown). Of the eight (10%) episodes with a bacteraemia, three were gram-positive and five were gram negative. Two FN episodes were admitted to ICU (at Days 1 and 11), one of which was diagnosed with severe sepsis.

**Table 2:** Demographic and outcome data of FN episodes.

	FN episodes without blood sample (n = 473)	FN episodes with blood sample at Day 1 (n = 79)	P- value
**Median age, years (IQR)**	4.3 (2.9–8.4)	6.0 (3.4–11.3)	0.043
**Female, n (%)**	223 (47.2)	39 (49.4)	0.717
**Diagnosis, n (%)**			
**–Haematological malignancy**	260 (55.0)	49 (62.0)	0.271
**–Solid tumour**	213 (45.0)	30 (38.0)	
**Severe sepsis at presentation, n (%)**	4 (0.9)	1 (1.3)	0.548
**ICU admission, n (%)**	15 (3.2)	2 (2.5)	>0.99
**Infection diagnosis**			
**–Bacteraemia**	66 (14.0)	8* (10.1)	0.14
**–Other MDI**	90 (19.0)	22 (27.8)	
**–CDI**	44 (9.3)	11 (13.9)	
**–FUO**	273 (57.7)	38 (48.1)	
**Median duration of antibiotics, days (IQR)**	6.3 (2.8–12.2)	4.9 (2.5–9.1)	0.04
**Median hospital LOS, days (IQR)**	6.0 (3.1–13.3)	5.13 (3.0-7.6)	0.07
**Died within 30 days**	2 (0.4)	0	>0.99

FN, febrile neutropenia; IQR, interquartile range; ICU, intensive care unit; MDI, microbiologically defined infection; CDI, clinically defined infection; FUO, fever of unknown cause; LOS, length of stay.

*Gram negative (Klebsiella pneumoniae/Pseudomonas aeruginosa, Escherichia fergusonii, Neisseria lactamica, Pseudomonas species, Escherichia coli) and Gram positive (Staphylococcus epidermidis, Enterococcus faecium, Enterococcus faecalis).

### Plasma Cytokine, CRP and PCT Profiles at FN Presentation

Plasma CRP and PCT levels as well as the abundance of 33 plasma cytokines and chemokines from 79 FN episodes with blood taken at time of FN presentation (Day 1) were quantified. The mean PCT (p = 0.0016), IL-10 (p = 0.0041) and Mip1-beta (p = 0.0078) values were significantly higher in episodes with bacteraemia than those without bacteraemia at FN presentation (Day 1) (**Supplementary Table 1**). In contrast, mean values of CRP as well as all other cytokines and chemokines including IL-6 and IL-8 were not significantly different in episodes with and without bacteraemia (**Supplementary Table 1**). The area under the curve (AUC), optimal threshold values, sensitivity, specificity and positive and negative likelihood ratios for CRP, PCT and the cytokines IL-6, IL-8, IL-10 and MIP1-beta are summarized in **Table 3**.

**Table 3:** Performance of biomarkers to predict bacteraemia at FN presentation (Day 1).

	AUC	p-value	Threshold	Sensitivity% (95% CI)	Specificity% (95% CI)	Positive LR	Negative LR	Youden Index
**PCT**	0.842	0.002	>0.425 [ng/ml]	100 (68–100)	78 (67–86)	4.5	0	0.77
**IL-10**	0.826	0.003	> 16.66 [pg/ml]	75.0 (41–96)	82 (71–89)	4.2	0.3	0.57
**MIP1-beta**	0.780	0.010	> 52.79 [pg/ml]	63 (31–86)	90 (81–95)	6.3	0.4	0.53
**CRP**	0.695	0.073	>62.5 [ug/ml]	50 (22–79)	78 (67–86)	2.3	0.6	0.27
**IL-6**	0.625	0.249	>356 [pg/ml]	50 (22–79)	90 (81–95)	5	0.6	0.40
**IL-8**	0.666	0.127	>665.5 [pg/ml]	50 (22–79)	80 (70–88)	2.5	0.6	0.30

AUC, Area Under the Curve; CI, Confidence interval; LR, likelihood ratio.

### Additive Performance of Biomarkers and Clinical Variables at Presentation

**Figure 1:**
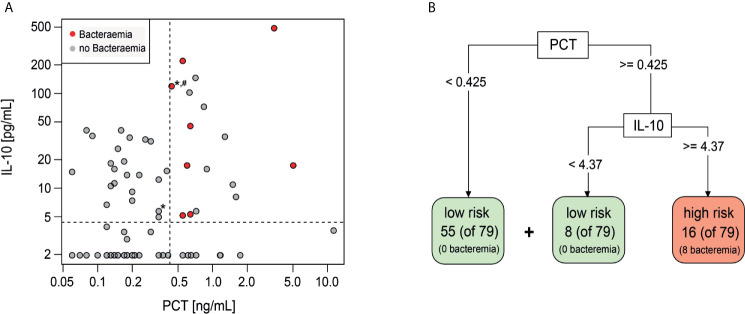
Decision tree classification model including PCT and IL-10. **(A)** Scatterplot showing distribution of IL-10 and PCT values of Day 1 samples. Red-bacteraemia cases, grey-no bacteraemia. (*) depicts ICU admission, (#) depicts severe sepsis diagnosis. **(B)** Decision tree model to identify low risk and high risk episodes using PCT and IL-10 with the indicated threshold values.

The additive performance of PCT, IL-10 and Mip1-beta was assessed in combination and together with individual clinical variables (platelets, maximum temperature and/or clinically unwell) that had been shown to predict bacteraemia in this cohort (13). Using a classification model based on a decision tree, the highest specificity and sensitivity was observed when combining PCT (threshold ≥0.425 ng/ml) and IL-10 (threshold ≥4.37 pg/ml) (**Figure 1A**). If this combination of biomarkers were used in isolation to risk stratify patients, 16 episodes would be classified as high-risk of which eight (50%) had a bacteraemia (**Figures 1A, B** **)**. Conversely, 63 (80%) episodes would be classified as low-risk, none of which had a bacteraemia. The combined PCT/IL-10 model had a sensitivity of 100% (95% CI 68.8–100%) and specificity of 89% (95% CI 80.0–95.0%) for prediction of bacteraemia. Cross-validation, which trains and assesses the predictor on independent data subsets, reduced the estimated sensitivity slightly to 87.5% (47.3–99.7%) and the specificity to 87.3% (77.3–94.0%). Of these 16 ‘high-risk’ episodes, one episode with bacteraemia (*Escherichia coli*) also had severe sepsis at FN presentation and was admitted to the ICU within 4.2 h. Of these 63 ‘low-risk’ episodes, one child was admitted to ICU with a late clinical deterioration, 11 days following initial FN presentation.

Adding clinical variables (platelets, maximum temperature and/or clinically unwell) into this classification model, either individually or in any combination, did not improve the predictive performance of the PCT and IL-10 biomarker combination.

The added diagnostic value of PCT and IL-10 to the AUS-rule CDR was also assessed. Using the AUS-rule in isolation and with a low-risk threshold set at ‘0,’ bacteraemia was correctly ruled out in only 10 (13%) FN episodes. Incorporating PCT (threshold <0.425 ng/ml = low risk) to the AUS-rule for scores of ‘1’ or above, a further 49 FN episodes (total 59, 75%) were correctly classified as ‘low-risk’ (**Table 4**). Incorporating both PCT and IL-10 to the AUS-rule for scores of ‘1’ or above, a further 7 FN episodes (total 66, 84%) were correctly classified as ‘low-risk’ as compared to using the AUS-rule and PCT alone. Similar to the combined PCT and IL-10 decision tree above, the FN episode without a bacteraemia but with a late onset clinical deterioration (day 11) would be classified as low risk using this approach.

**Table 4:** Combined performance of AUS-rule and PCT and IL-10 for prediction of bacteraemia episodes in children with febrile neutropenia (shaded area are episodes classified as low-risk).

		Risk status according to AUS-rule				
		Low risk	High Risk			Total, n (%)
		0 (n = 10)	1 (n = 26)	2 (n = 28)	3 (n = 15)	
**Risk status according to PCT and/or IL-10**	**Procalcitonin**					
	Low risk (PCT <0.425 ng/ml)—no bacteraemia, n	6	21	17	11*	55 (69.6%)
	High risk (PCT ≥0.425 ng/ml)—no bacteraemia, n	4	3	9	0	16 (20.3%)
	High risk (PCT ≥0.425 ng/ml), bacteraemia, n	0	2	2	4^	8 (10.1%)
	**Procalcitonin and Interleukin-10**					
	Low risk (PCT <0.425 ng/ml), no bacteraemia, n	6	21	17	11*	55 (69.6%)
	Low risk (PCT ≥0.425 ng/ml & IL-10 <4.37 pg/ml), no bacteraemia, n	1	1	6	0	8 (10.1%)
	High risk (PCT ≥0.425 ng/ml & IL-10 ≥4.37 pg/ml), no bacteraemia, n	3	2	3	0	8 (10.1%)
	High risk (PCT ≥0.425 ng/ml & IL-10 ≥4.37 pg/ml), bacteraemia, n	0	2	2	4^	8 (10.1%)

*includes one episode admitted to ICU 11 days after initial presentation with FN; ^ includes one episode with severe E. coli bacteraemia, sepsis at presentation and ICU admission.

### Plasma Cytokine, CRP and PCT Profiles at Day 2

Plasma levels of CRP, PCT and 33 cytokines and chemokines from 31 of the 79 episodes that had a repeat blood sample taken at Day 2 (8–24 h from presentation) were available for analysis (**Supplementary Table 2**). At Day 2, PCT (p = 0.018), IFN-alpha (p = 0.0271) and IL-23 (p = 0.0292) were able to distinguish bacteraemia cases from all other underlying causes of FN with statistical significance (**Supplementary Tables 2** and **3**).

### Comparison of Biomarker Dynamics Between FN Presentation (Day 1) and Day 2

**Figure 2:**
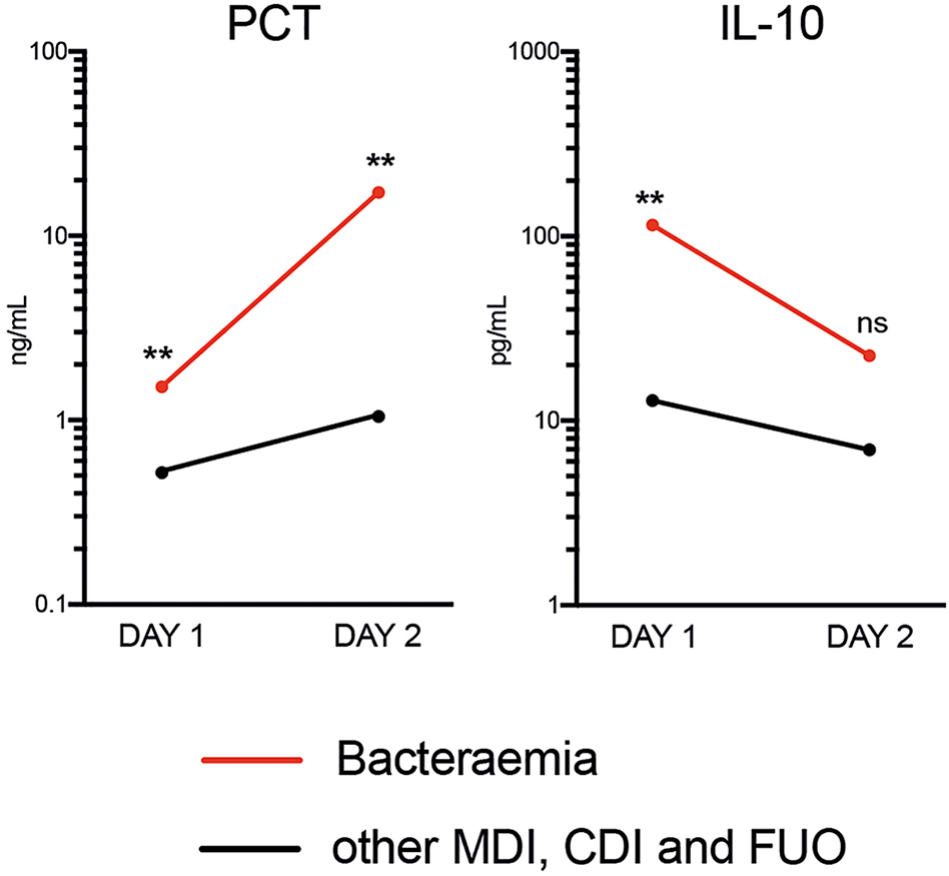
Mean Plasma PCT and IL-10 levels between time of FN presentation (Day 1) and Day 2 when comparing bacteraemia cases with all other causes of FN. P-values shown were calculated using ROC analysis comparing bacteraemia cases with non-bacteraemia cases at Days 1 and 2. Data shown as Mean and SEM. **p <0.005, ^ns^p ^>^0.05 (not significant).

Only PCT was able to distinguish bacteraemia and non-bacteraemia FN episodes with statistical significance at both time points (**Table 3** and **Supplementary Table 3**). When comparing the kinetics of plasma PCT levels in the bacteraemia group, an increase by >11-fold from Days 1 to 2 (**Figure 2**) was identified. Mean CRP levels also increased, albeit at lower amplitude (2-fold) (see **Supplementary Figure 1**). In contrast, plasma levels of all 33 analysed cytokines decreased in bacteraemia cases at Day 2 compared to Day 1 by an average of 3.6-fold, including IL-10 (5-fold), MIP1-beta (7.1-fold), IL-6 (6.1-fold), IL-8 (2.4-fold), IFN-alpha (7.7-fold) and IL-23 (2.8-fold) (**Figure 2** and **Supplementary Figure 1**). In all non-bacteraemia cases, only a subtle increase in PCT (2-fold) or CRP (1.8-fold) was identified between Days 1 and 2, as well as only minor decreases in the levels of all cytokines and chemokines tested (average decrease of 1.2-fold) with IL-1ra (2-fold decrease) showing the biggest difference between the two time points (**Figure 2**, **Supplementary Figure 1** and **Supplementary Table 4**).

## Discussion

Early diagnosis of bacteraemia in children with cancer and FN remains a challenge for modern medicine due to the lack of highly sensitive and specific laboratory markers and clinical signs. This multi-site, prospective study has identified three biomarkers, IL-10, Mip1-beta as well as the commercially available PCT, that were able to differentiate children with FN who had a documented bacteraemia from those who did not at initial hospital presentation. The combination of an elevated PCT and IL-10 had a sensitivity of 100% and specificity of 89% for the prediction of initial bacteraemia, correctly identifying all bacteraemia episodes and classifying 80% of episodes as low risk and 20% as high risk. This result is superior to most published paediatric FN CDRs (3). Furthermore, the addition of PCT and IL-10 to an existing FN CDR (‘AUS-rule’) increased the ability of this rule to classify low-risk FN episodes by more than six-fold (13). These data suggest that biomarkers such as PCT and IL-10 may have a role in enhancing existing CDRs or risk-stratification strategies in children with cancer and FN.

Consistent with our data, several studies have identified PCT as a more reliable biomarker compared to CRP for the prediction of bacterial infection, both at FN presentation as well as in serial measurements (11). Procalcitonin, which reaches plateau values within 8–24 h, has been shown to be a useful marker for early diagnosis of sepsis in adult patients as well as differentiation of bacterial infection from other causes of inflammation, including viral infection (18, 20). More recently, studies investigating PCT-guided antibiotic discontinuation algorithms in predominantly critically unwell adults, indicate it may be safe, cost-effective and reduce antibiotic duration in selected patient populations (20, 21). While there are at least 22 studies investigating PCT in children with cancer and FN, it is not routinely used in practice, nor has been incorporated into published risk stratification strategies (11). Heterogeneity in laboratory techniques and outcome definitions, as well as small sample sizes may, in part, explain this and could be overcome by individual participant data metanalyses as have been successfully used in other areas of FN research (2) Prospective validation and implementation studies are urgently required to determine the diagnostic role of PCT in the FN population.

Interleukin-10, an anti-inflammatory cytokine, has a crucial role in limiting the immune response to infection thereby preventing inflammation and excessive damage to the host (22) Several groups have previously identified prognostic values for IL-10 for prediction of bacteraemia, either on its own in FN in children (23–26) or in combination with PCT in FN in adult patients (27). However, the vast majority of these data are generated from small, single centre studies that have investigated the various markers in isolation. MIP-1beta, a chemoattractant for a wide range of immune cells, has also been previously identified as potential biomarker to predict survival in paediatric septic shock (28) and our study confirms a potential usefulness for this and IL-10 to be included in future biomarker studies in FN.

C-reactive protein did not reliably differentiate FN episodes with and without bacteraemia, either at presentation or Day 2, in our study. This is consistent with previous studies that show that CRP as biomarker for serious infections performs poorly in FN (11). However, despite this, the marker has been used successfully in paediatric FN risk-prediction models (29, 30). Interleukin-6 and IL-8 were also non-discriminatory for the prediction bacteraemia in this population. This is in contrast to other paediatric FN studies, including a FN CDR that incorporates IL-8 to differentiate patients at high and low-risk of infection (11, 31). Interleukin-8 has previously also been demonstrated to rise rapidly in the presence of severe infection (32) and IL-6 has been proposed as a marker of life-threatening gram-negative bacterial infections (33) Different outcomes used, as well as the low number of gram-negative infections in our group may, in part, explain this difference.

Just as clinical symptoms or signs are rarely used in isolation to predict infection or adverse outcome, a combination of different biomarkers may have a role. In our study, all cases of bacteraemia were detected in FN episodes with elevated PCT and IL-10. However, while this was statistically validated using a leave-one-out technique, the overall sample size and proportion with bacteraemia were small and prospective validation is essential. The combination of an elevated PCT and IL-10, albeit at different thresholds, was similarly shown to have a better predictive ability than the individual markers in 100 adult patients with haematological malignancy (27). The clinical impact of using biomarker combinations for risk stratification is significant, with up to 80% of FN episodes identified as low-risk in our cohort based on PCT and IL-10 results. These patients are potentially eligible for reduced intensity FN treatment including home-based care and oral antibiotics. Using a structured FN pathway incorporating safety-nets, risk-stratification has been shown to be safe and significantly reduce hospital length of stay, antibiotic exposure and costs of care and improve quality of life (6). While our study has found that biomarker combinations may be more accurate than existing CDRs and identify a larger pool of low risk patients, further studies in addition to validation, are required to explore feasibility and cost of this approach.

The additive performance of novel biomarkers to routinely available clinical and laboratory parameters in children with FN remains unknown. Looking specifically at the clinical variables shown to predict bacterial infection in the larger Australian PICNICC study cohort (platelets, elevated temperature and clinically unwell) we observed no additional benefit to the high sensitivity and specificity PCT and IL-10 combination (13). A small benefit was observed when adding PCT and IL-10 to the AUS-rule, as compared to the PCT and IL-10 combination alone, with 66 (84%) episodes correctly classified as low-risk compared to 63 (80%) episodes. This is of particular interest, as the AUS-rule has been validated internationally and recently implemented in Australia (15, 19) to predict bacterial infection in children with cancer and FN.

Analysis of blood samples collected at Day 2 enabled comparison of biomarkers kinetics. While both PCT and CRP levels increased in patients with bacteraemia, aligning with previous reports, a significant difference was only detected for PCT (13). Interestingly, the overall cytokine levels on Day 2 decreased significantly in bacteraemia episodes compared to the only subtle decreases observed in the non-bacteraemia episodes. This may be reflective of a response to antibiotic therapy for patients with a confirmed bacterial infection and a lack of response to antibiotic therapy for non-infective or viral causes of fever. The role of cytokines for monitoring treatment response in FN warrant further evaluation.

Our prospective, two-centre study provides further insights into the potential clinical utility of biomarkers, such as the routinely available PCT, in the paediatric FN risk stratification process. While our results show some consistencies with previously published data, our sample size did not permit detailed analysis of impact of gram-negative versus gram-positive infection. Furthermore, the lower number of Day 2 samples available for analysis, limit the conclusion that can be drawn about biomarker kinetics. While we included additional clinical outcome data such as sepsis and ICU admissions, the number of these adverse events is also low. Reassuringly, biomarkers were elevated in the patient with bacteraemia and severe sepsis at presentation requiring ICU-level care. However, while the second ICU admission occurring at 11 days was not associated with an elevated PCT at presentation, the cause of this late clinical deterioration is likely a new event and would not be expected to be predicted at FN onset. This highlights the need for any FN risk-stratification process and subsequent reduced intensity treatment for low-risk patients to include additional safeguards and clear pathways for readmission or re-escalation of care.

Currently no paediatric FN CDR can perfectly predict infections or adverse outcomes in children presenting with FN (16). Our data generated in a limited cohort of patients supports the additive value of blood biomarker molecules including PCT both at presentation and during treatment response. Before consideration for routine clinical use, prospective validation is necessary as well as detailed consideration as to the feasibility and cost-effectiveness of using this approach.

## Data Availability Statement

The raw data supporting the conclusions of this article will be made available by the authors, without undue reservation.

## Ethics Statement

The studies involving human participants were reviewed and approved by HREC/16/RCHM/108. Written informed consent to participate in this study was provided by the participants’ legal guardian/next of kin.

## Author Contributions

MD scoped parts of the project and designed, performed, and analysed experiments. GH scoped the project and analysed experiments. MP and KT scoped the project and designed experiments. CL-W-S and GS analysed experiments. MD, GH, MP, and KT wrote the manuscript with input from all co-authors. All authors contributed to the article and approved the submitted version.

## Funding

This study was funded by a National Health and Medical Research Council (NHMRC) Project Grant (APP1104527). We gratefully acknowledge the support and endorsement of the Australian and New Zealand Children’s Haematology/Oncology Group (ANZCHOG) and the Paediatric Research in Emergency Departments International Collaborative (PREDICT). 

## Conflict of Interest

The authors declare that the research was conducted in the absence of any commercial or financial relationships that could be construed as a potential conflict of interest.
