# Supplementary material for: Procalcitonin and Interleukin-10 May Assist in Early Prediction of Bacteraemia in Children With Cancer and Febrile Neutropenia
Source: Front Immunol. 2021 May 20;12:641879. doi: 10.3389/fimmu.2021.641879 (PMC8173204; doi:10.3389/fimmu.2021.641879)
Supplement: Supplementary file 1 [file DataSheet_1.docx]

**SUPPLEMENTAL MATERIAL**

**Procalcitonin and Interleukin-10 may assist in early prediction of bacteraemia in children with cancer and febrile neutropenia.**

Marcel Doerflinger^*^ and Gabrielle M Haeusler^*^ *et al.*

**Supplementary Table 1:** Plasma CRP, PCT, Cytokine and Chemokine values at time of FN presentation

| **Day 1** | **bacteraemia (N=8)** | | **no bacteraemia (N=71)** | |
| --- | --- | --- | --- | --- |
|  | Mean | SEM | Mean | SEM |
| **CRP [mg/L]** | 77.63 | 25.88 | 43.08 | 5.87 |
| **PCT [ng/mL]** | 1.49 | 0.62 | 0.51 | 0.16 |
| **Eotaxin [pg/mL]** | 152.11 | 84.32 | 115.48 | 20.47 |
| **GM-CSF [pg/mL]** | 84.72 | 59.13 | 46.70 | 7.99 |
| **GRO-alpha [pg/mL]** | 427.03 | 82.59 | 349.85 | 40.39 |
| **IFNa [pg/mL]** | 7.17 | 2.62 | 5.33 | 0.64 |
| **IFNg [pg/mL]** | 676.43 | 156.69 | 742.69 | 81.31 |
| **IL1a [pg/mL]** | 7.49 | 2.87 | 6.40 | 0.98 |
| **IL1b [pg/mL]** | 10.93 | 3.92 | 11.67 | 1.93 |
| **IL1ra [pg/mL]** | 1213.99 | 430.53 | 962.99 | 176.94 |
| **IL10 [pg/mL]** | 112.35 | 60.01 | 13.22 | 2.81 |
| **IL12p70 [pg/mL]** | 22.16 | 7.49 | 12.90 | 1.53 |
| **IL13 [pg/mL]** | 16.04 | 7.33 | 12.61 | 1.76 |
| **IL15 [pg/mL]** | 39.16 | 19.43 | 41.18 | 7.71 |
| **IL17a [pg/mL]** | 25.80 | 13.55 | 24.24 | 9.38 |
| **IL18 [pg/mL]** | 562.18 | 92.33 | 480.27 | 46.14 |
| **IL2 [pg/mL]** | 47.56 | 36.16 | 17.52 | 2.73 |
| **IL21 [pg/mL]** | 508.68 | 203.87 | 425.33 | 76.39 |
| **IL22 [pg/mL]** | 239.98 | 105.96 | 152.00 | 29.48 |
| **IL23 [pg/mL]** | 432.60 | 146.26 | 473.76 | 49.84 |
| **IL27 [pg/mL]** | 447.53 | 236.34 | 448.28 | 68.17 |
| **IL31 [pg/mL]** | 41.52 | 21.65 | 49.59 | 10.14 |
| **IL4 [pg/mL]** | 280.54 | 149.57 | 282.50 | 42.84 |
| **IL5 [pg/mL]** | 66.29 | 53.01 | 21.66 | 2.98 |
| **IL6 [pg/mL]** | 623.45 | 380.79 | 169.10 | 17.74 |
| **IL7 [pg/mL]** | 19.84 | 11.34 | 11.65 | 1.37 |
| **IL8 [pg/mL]** | 690.01 | 236.38 | 420.88 | 48.36 |
| **IL9 [pg/mL]** | 145.63 | 53.08 | 114.09 | 23.08 |
| **IP10 [pg/mL]** | 2180.70 | 737.16 | 3585.43 | 550.56 |
| **MCP1 [pg/mL]** | 3007.29 | 1030.77 | 1578.82 | 130.68 |
| **MIP1a [pg/mL]** | 62.47 | 18.78 | 45.87 | 6.12 |
| **MIP1b [pg/mL]** | 124.32 | 72.89 | 19.70 | 4.08 |
| **RANTES [pg/mL]** | 354.05 | 159.62 | 625.75 | 64.89 |
| **SDF1a [pg/mL]** | 4908.26 | 1113.20 | 6394.38 | 573.70 |
| **TNFa [pg/mL]** | 13.85 | 3.60 | 14.39 | 2.88 |

**Supplementary Table 2:** Plasma CRP, PCT, Cytokine and Chemokine values at Day 2

| **Day 2** | **bacteraemia (N=4)** | | **no bacteraemia (N=27)** | |
| --- | --- | --- | --- | --- |
|  | Mean | SEM | Mean | SEM |
| **CRP [mg/L]** | 153.50 | 47.39 | 78.56 | 14.89 |
| **PCT [ng/mL]** | 16.94 | 14.50 | 1.03 | 0.49 |
| **Eotaxin [pg/mL]** | 30.49 | 6.41 | 79.89 | 13.49 |
| **GM-CSF [pg/mL]** | 17.16 | 3.48 | 41.46 | 7.89 |
| **GRO-alpha [pg/mL]** | 100.38 | 42.84 | 310.36 | 68.43 |
| **IFNa [pg/mL]** | 0.93 | 0.29 | 4.92 | 0.89 |
| **IFNg [pg/mL]** | 309.36 | 92.46 | 554.87 | 108.94 |
| **IL1a [pg/mL]** | 5.02 | 3.68 | 6.17 | 1.26 |
| **IL1b [pg/mL]** | 3.32 | 0.68 | 8.23 | 1.41 |
| **IL1ra [pg/mL]** | 406.89 | 256.34 | 491.47 | 82.95 |
| **IL10 [pg/mL]** | 22.50 | 20.54 | 9.15 | 2.08 |
| **IL12p70 [pg/mL]** | 7.93 | 2.52 | 9.12 | 1.79 |
| **IL13 [pg/mL]** | 5.44 | 0.00 | 10.54 | 1.83 |
| **IL15 [pg/mL]** | 8.16 | 4.09 | 32.72 | 7.49 |
| **IL17a [pg/mL]** | 7.85 | 4.45 | 29.15 | 18.68 |
| **IL18 [pg/mL]** | 379.55 | 109.51 | 387.66 | 57.87 |
| **IL2 [pg/mL]** | 10.02 | 4.19 | 13.27 | 2.40 |
| **IL21 [pg/mL]** | 334.91 | 295.47 | 379.59 | 115.22 |
| **IL22 [pg/mL]** | 97.86 | 71.18 | 133.30 | 34.45 |
| **IL23 [pg/mL]** | 156.70 | 80.45 | 484.16 | 60.84 |
| **IL27 [pg/mL]** | 96.95 | 30.43 | 334.97 | 75.15 |
| **IL31 [pg/mL]** | 23.16 | 5.25 | 48.69 | 12.33 |
| **IL4 [pg/mL]** | 54.76 | 45.00 | 231.35 | 49.76 |
| **IL5 [pg/mL]** | 14.95 | 5.43 | 20.87 | 4.18 |
| **IL6 [pg/mL]** | 101.94 | 30.57 | 151.29 | 23.23 |
| **IL7 [pg/mL]** | 5.82 | 3.73 | 8.35 | 1.85 |
| **IL8 [pg/mL]** | 291.35 | 71.18 | 378.36 | 78.86 |
| **IL9 [pg/mL]** | 38.90 | 20.91 | 94.17 | 27.61 |
| **IP10 [pg/mL]** | 391.54 | 65.90 | 3201.95 | 973.42 |
| **MCP1 [pg/mL]** | 1397.76 | 482.83 | 1132.23 | 151.87 |
| **MIP1a [pg/mL]** | 20.12 | 7.01 | 38.19 | 8.21 |
| **MIP1b [pg/mL]** | 17.39 | 7.51 | 17.96 | 6.32 |
| **RANTES [pg/mL]** | 135.53 | 47.75 | 673.01 | 142.11 |
| **SDF1a [pg/mL]** | 3512.30 | 2010.02 | 5106.05 | 938.63 |
| **TNFa [pg/mL]** | 12.47 | 4.70 | 14.31 | 1.80 |

**Supplementary Table 3**: Performance of biomarkers to predict bacteraemia at Day 2

|  | **AUC** | **p-value** | **Threshold** | **Sensitivity% (95% CI)** | **Specificity%**  **(95% CI)** | **Positive LR** | **Negative LR** | **Youden Index** |
| --- | --- | --- | --- | --- | --- | --- | --- | --- |
| **PCT** | 0.8704 | 0.018 | > 0.540 [ng/mL] | 100  (50-100) | 74  (55-87) | 3.8 | 0 | 0.74 |
| **IFN-alpha** | 0.847 | 0.027 | >0.79  [pg/mL] | 75  (30-99) | 82  (63-91) | 4.2 | 0.3 | 0.56 |
| **IL-23** | 0.843 | 0.029 | >54.25 [pg/mL] | 50  (9-91) | 96  (82-100) | 12.5 | 0.5 | 0.46 |
| **CRP** | 0.792 | 0.063 | > 176 [ug/mL] | 50  (9-91) | 85  (68-94) | 5 | 0.4 | 0.35 |
| **IL-6** | 0.583 | 0.596 | >131.5 [pg/mL] | 75  (30-99) | 56  (37-72) | 1.7 | 0.4 | 0.31 |
| **IL-8** | 0.500 | >0.999 | >271.4  [pg/mL] | 75  (30-99) | 56  (37-72) | 1.3 | 0.6 | 0.31 |
| **IL-10** | 0.542 | 0.791 | >2.77  [pg/mL] | 75  (30-99) | 44  (28-63) | 2.3 | 0.6 | 0.19 |
| **MIP1-beta** | 0.602 | 0.517 | >15.54 [pg/mL] | 50  (9-91) | 78  (59-89) | 2.3 | 0.6 | 0.28 |

AUC, Area Under the Curve; CI, Confidence interval; LR, likelihood ratio

**Supplementary Table 4**: Plasma CRP, PCT, Cytokine and Chemokine value comparison between Day1 and Day 2

| **bacteraemia** | **mean DAY1 (N=8)** | **mean DAY2 (N=4)** | **fold increase** | **fold decrease** | **No bacteraemia** | **mean DAY1 (N=71)** | **mean DAY2 (N=27)** | **fold increase** | **fold decrease** |
| --- | --- | --- | --- | --- | --- | --- | --- | --- | --- |
| **CRP [mg/L]** | 77.63 | 153.50 | 2.0 | 0.5 | **CRP [mg/L]** | 43.08 | 78.56 | 1.8 | 0.5 |
| **PCT [ng/mL]** | 1.49 | 16.94 | 11.4 | 0.1 | **PCT [ng/mL]** | 0.51 | 1.03 | 2.0 | 0.5 |
| **Eotaxin [pg/mL]** | 152.11 | 30.49 | 0.2 | 5.0 | **Eotaxin [pg/mL]** | 115.48 | 79.89 | 0.7 | 1.4 |
| **GM-CSF [pg/mL]** | 84.72 | 17.16 | 0.2 | 4.9 | **GM-CSF [pg/mL]** | 46.70 | 41.46 | 0.9 | 1.1 |
| **GRO-alpha [pg/mL]** | 427.03 | 100.38 | 0.2 | 4.3 | **GRO-alpha [pg/mL]** | 349.85 | 310.36 | 0.9 | 1.1 |
| **IFNa [pg/mL]** | 7.17 | 0.93 | 0.1 | 7.7 | **IFNa [pg/mL]** | 5.33 | 4.92 | 0.9 | 1.1 |
| **IFNg [pg/mL]** | 676.43 | 309.36 | 0.5 | 2.2 | **IFNg [pg/mL]** | 742.69 | 554.87 | 0.7 | 1.3 |
| **IL1a [pg/mL]** | 7.49 | 5.02 | 0.7 | 1.5 | **IL1a [pg/mL]** | 6.40 | 6.17 | 1.0 | 1.0 |
| **IL1b [pg/mL]** | 10.93 | 3.32 | 0.3 | 3.3 | **IL1b [pg/mL]** | 11.67 | 8.23 | 0.7 | 1.4 |
| **IL1ra [pg/mL]** | 1213.99 | 406.89 | 0.3 | 3.0 | **IL1ra [pg/mL]** | 962.99 | 491.47 | 0.5 | 2.0 |
| **IL10 [pg/mL]** | 112.35 | 22.50 | 0.2 | 5.0 | **IL10 [pg/mL]** | 13.22 | 9.15 | 0.7 | 1.4 |
| **IL12p70 [pg/mL]** | 22.16 | 7.93 | 0.4 | 2.8 | **IL12p70 [pg/mL]** | 12.90 | 9.12 | 0.7 | 1.4 |
| **IL13 [pg/mL]** | 16.04 | 5.44 | 0.3 | 2.9 | **IL13 [pg/mL]** | 12.61 | 10.54 | 0.8 | 1.2 |
| **IL15 [pg/mL]** | 39.16 | 8.16 | 0.2 | 4.8 | **IL15 [pg/mL]** | 41.18 | 32.72 | 0.8 | 1.3 |
| **IL17a [pg/mL]** | 25.80 | 7.85 | 0.3 | 3.3 | **IL17a [pg/mL]** | 24.24 | 29.15 | 1.2 | 0.8 |
| **IL18 [pg/mL]** | 562.18 | 379.55 | 0.7 | 1.5 | **IL18 [pg/mL]** | 480.27 | 387.66 | 0.8 | 1.2 |
| **IL2 [pg/mL]** | 47.56 | 10.02 | 0.2 | 4.7 | **IL2 [pg/mL]** | 17.52 | 13.27 | 0.8 | 1.3 |
| **IL21 [pg/mL]** | 508.68 | 334.91 | 0.7 | 1.5 | **IL21 [pg/mL]** | 425.33 | 379.59 | 0.9 | 1.1 |
| **IL22 [pg/mL]** | 239.98 | 97.86 | 0.4 | 2.5 | **IL22 [pg/mL]** | 152.00 | 133.30 | 0.9 | 1.1 |
| **IL23 [pg/mL]** | 432.60 | 156.70 | 0.4 | 2.8 | **IL23 [pg/mL]** | 473.76 | 484.16 | 1.0 | 1.0 |
| **IL27 [pg/mL]** | 447.53 | 96.95 | 0.2 | 4.6 | **IL27 [pg/mL]** | 448.28 | 334.97 | 0.7 | 1.3 |
| **IL31 [pg/mL]** | 41.52 | 23.16 | 0.6 | 1.8 | **IL31 [pg/mL]** | 49.59 | 48.69 | 1.0 | 1.0 |
| **IL4 [pg/mL]** | 280.54 | 54.76 | 0.2 | 5.1 | **IL4 [pg/mL]** | 282.50 | 231.35 | 0.8 | 1.2 |
| **IL5 [pg/mL]** | 66.29 | 14.95 | 0.2 | 4.4 | **IL5 [pg/mL]** | 21.66 | 20.87 | 1.0 | 1.0 |
| **IL6 [pg/mL]** | 623.45 | 101.94 | 0.2 | 6.1 | **IL6 [pg/mL]** | 169.10 | 151.29 | 0.9 | 1.1 |
| **IL7 [pg/mL]** | 19.84 | 5.82 | 0.3 | 3.4 | **IL7 [pg/mL]** | 11.65 | 8.35 | 0.7 | 1.4 |
| **IL8 [pg/mL]** | 690.01 | 291.35 | 0.4 | 2.4 | **IL8 [pg/mL]** | 420.88 | 378.36 | 0.9 | 1.1 |
| **IL9 [pg/mL]** | 145.63 | 38.90 | 0.3 | 3.7 | **IL9 [pg/mL]** | 114.09 | 94.17 | 0.8 | 1.2 |
| **IP10 [pg/mL]** | 2180.70 | 391.54 | 0.2 | 5.6 | **IP10 [pg/mL]** | 3585.43 | 3201.95 | 0.9 | 1.1 |
| **MCP1 [pg/mL]** | 3007.29 | 1397.76 | 0.5 | 2.2 | **MCP1 [pg/mL]** | 1578.82 | 1132.23 | 0.7 | 1.4 |
| **MIP1a [pg/mL]** | 62.47 | 20.12 | 0.3 | 3.1 | **MIP1a [pg/mL]** | 45.87 | 38.19 | 0.8 | 1.2 |
| **MIP1b [pg/mL]** | 124.32 | 17.39 | 0.1 | 7.1 | **MIP1b [pg/mL]** | 19.70 | 17.96 | 0.9 | 1.1 |
| **RANTES [pg/mL]** | 354.05 | 135.53 | 0.4 | 2.6 | **RANTES [pg/mL]** | 625.75 | 673.01 | 1.1 | 0.9 |
| **SDF1a [pg/mL]** | 4908.26 | 3512.30 | 0.7 | 1.4 | **SDF1a [pg/mL]** | 6394.38 | 5106.05 | 0.8 | 1.3 |
| **TNFa [pg/mL]** | 13.85 | 12.47 | 0.9 | 1.1 | **TNFa [pg/mL]** | 14.39 | 14.31 | 1.0 | 1.0 |
|  |  | **Average change DAY1 vs DAY2:** | **0.4** | **3.6** |  |  | **Average change DAY1 vs DAY2:** | **0.8** | **1.2** |


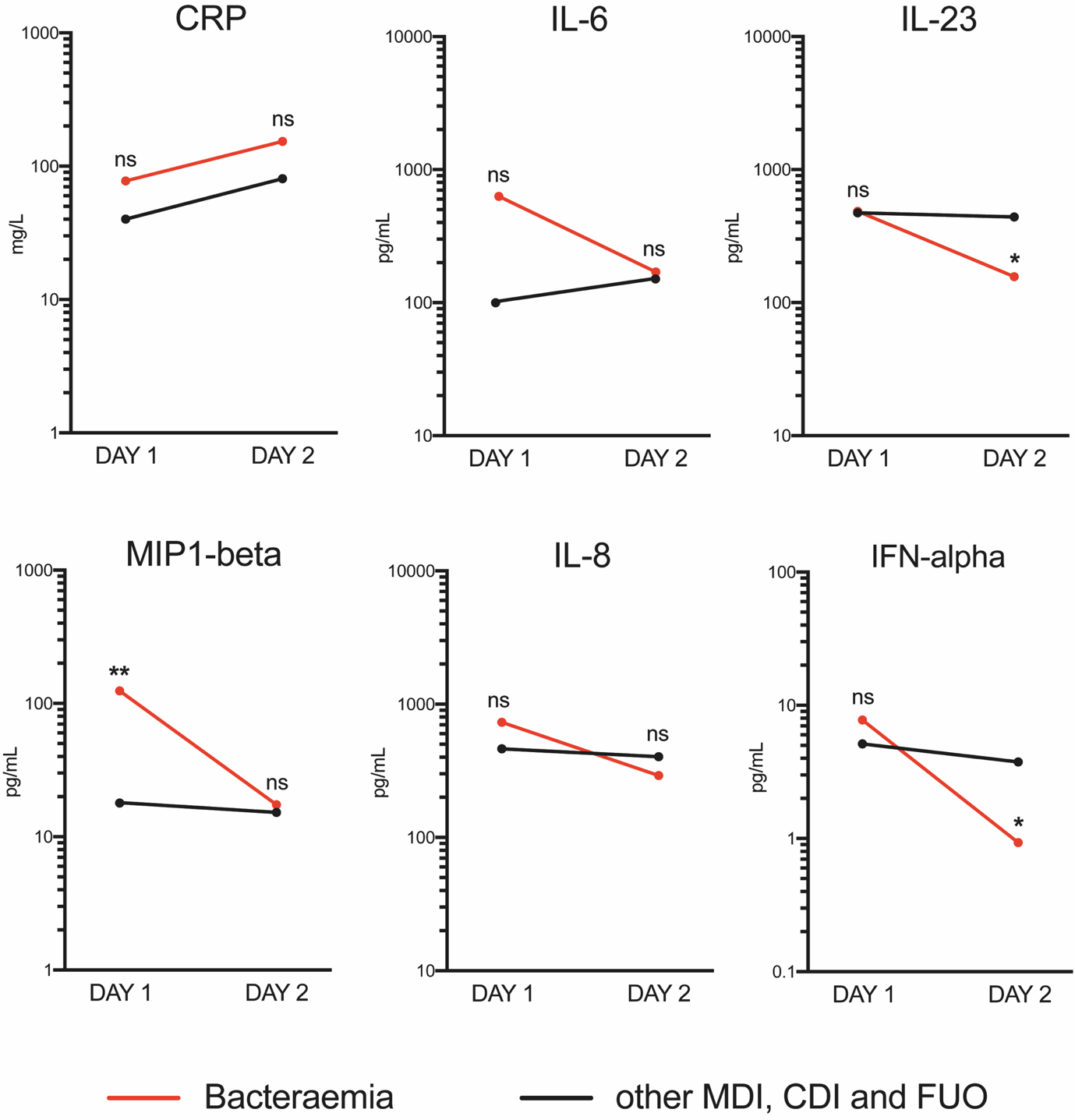


**Supplementary Figure 1:** Mean Plasma CRP and Cytokine levels between time of FN presentation (Day 1) and Day 2 when comparing bacteraemia cases with all other causes of FN. P-values shown were calculated using ROC analysis comparing bacteraemia cases with non-bacteraemia cases at Day 1 and Day 2. Data shown as Mean and SEM. **p<0.005, *p<0.05, ^ns^p>0.05 (not significant).
